# Unraveling Oxidative Threads: Stress, Pathology, and Resilience in Biochemistry, Molecular, and Cellular Biology (2023–2025)

**DOI:** 10.3390/cimb47121050

**Published:** 2025-12-16

**Authors:** Stuart Maudsley

**Affiliations:** Receptor Biology Lab., Department of Drug Discovery, H. Lee Moffitt Cancer Center, 12902 Magnolia Drive, Tampa, FL 33612, USA; stuart.maudsley@moffitt.org

The years 2023–2025 have generated a profound evolution in biochemistry and molecular and cellular biology, where the threads of oxidative stress weave through aging, environmental insults, and human disease, and—increasingly—therapeutic opportunity. These years have intensified scrutiny of molecular pathologies and illuminated how reactive oxygen species (ROS) and stress-responsive pathways—hallmarks of my own research [1,2,3]—drive cellular dysfunction across reproductive, vascular, metabolic, and neural systems. A landmark 2023 *Current Issues in Molecular Biology* (*CIMB*) review on sperm cryopreservation exemplifies this paradigm, showing how freezing–thawing cycles trigger osmotic, thermal, and oxidative insults that elevate ROS, impair sperm motility and viability, and provoke lipid peroxidation and mitochondrial collapse [4]. This process strikingly recapitulates age-associated fertility decline and chemotherapy-induced gonadal damage, where cumulative oxidative injury erodes chromatin integrity and fertilization competence. Complementing this reproductive angle, a 2025 *CIMB* article dissects oxidative stress in erectile dysfunction (ED), identifying NADPH oxidase and uncoupled eNOS as primary ROS generators that scavenge nitric oxide, drive endothelial dysfunction, and culminate in corporal fibrosis [5]. Aging and comorbidities such as diabetes and hypertension amplify these cascades, positioning ED as an early clinical sentinel of systemic vascular aging. These reproductive and vascular stories converge dramatically in neuroinflammatory disease. A 2023 *CIMB* synthesis on multiple sclerosis reveals how ROS-fueled mitochondrial–ER stress loops, combined with Nrf2 and sirtuin dysregulation, drive demyelination, axonal transection, and maladaptive glial responses—processes markedly accelerated by aging [6]. Parallel 2025 *CIMB* reviews demonstrate that environmental micro(nano)plastics act as insidious ROS inducers, breaching gut barriers, reshaping the microbiota, and—via the gut–brain axis—provoking TLR4/NF-κB-driven neuroinflammation, microglial pyroptosis, amyloid-β accumulation, and α-synuclein propagation [7,8]. These findings recast modern pollutants as potent accelerators of neurodegenerative aging. Amid these pathologies, the 2023–2025 period has led to the creation of powerful countermeasures. A comprehensive 2025 *CIMB* compendium on flavonoids establishes these polyphenols as pleiotropic ROS scavengers that upregulate Nrf2/HO-1, suppress NF-κB/MAPK signaling, and restore insulin sensitivity even when IRS-1 and IRS-2 are disabled by stress-induced serine phosphorylation [9,10]. Quercetin, luteolin, and related compounds not only attenuate hepatic steatosis and β-cell death but also block ferroptosis in neural models and synergize with PDE5 inhibitors in ED. Their protective reach extends to the extracellular matrix: a 2025 *CIMB* analysis shows that hydrolyzed collagen peptides counteract UV- and glycation-induced cross-linking, preserving dermal resilience against oxidative fragmentation [11]. These tightly interlinked *CIMB* contributions are amplified by broader milestones: AlphaFold3-enabled docking of flavonoid–protein interactions [12], CRISPR-mediated Nrf2 augmentation in remyelination models [13], and single-cell multi-omics mapping of ROS gradients in microplastic-exposed tissues [14]. Challenges remain—flavonoid bioavailability, the ethical boundaries of germline or neural editing, and the largely unexplored synergy between microplastics and metabolic stress in the elderly—but the horizon is bright. By 2030, rapid advances in computational power and experimental models are expected to converge, opening entirely new avenues for redox biology and personalized anti-aging interventions. Quantum-enhanced reactive oxygen species (ROS) simulations leveraging quantum computing platforms will likely achieve unprecedented accuracy in modeling electron transfer and spin states during oxidative events, overcoming the current limitations of classical molecular dynamics that struggle with the multi-reference character of ROS chemistry [15,16,17]. Simultaneously, exposome-guided preventive strategies will mature as large-scale longitudinal cohorts (e.g., the Human Exposome Project and NIH All of Us initiative) integrate real-time environmental, dietary, and lifestyle data with multi-omics profiling. Machine learning frameworks trained on these datasets are already predicting individual oxidative burden with >85% accuracy [18], paving the way for pre-symptomatic deployment of targeted flavonoid-rich interventions that minimize cumulative ROS-induced damage long before clinical manifestations appear. Finally, the emergence of stress-resilient organoids—engineered cerebral, cardiac, and dermal organoids subjected to controlled oxidative and glycative stress—will serve as patient-specific platforms for testing flavonoid–collagen protective axes. Recent breakthroughs in vascularized and innervated organoids [19] and CRISPR-based enhancement of endogenous antioxidant pathways such as Nrf2 and SIRT1 [20] suggest that, by the end of the decade, we will be able to generate “aged” organoids that faithfully recapitulate an individual’s lifetime ROS exposure, then rapidly screen bespoke polyphenol–collagen regimens to restore extracellular matrix integrity and tissue function [21]. Together, these three converging technologies—quantum redox modeling, exposome-driven prediction, and resilient personalized organoids—promise to shift the paradigm from the reactive treatment of age-related disease to proactive, mechanistically precise prevention at the molecular and tissue levels [22]. *CIMB*’s 2023–2025 stress-centric publications, collectively exceeding 50,000 views, testify to the vitality of this domain and its power to convert molecular pathology into preventive and therapeutic opportunities. As custodians of these oxidative threads, we are called to weave ever-bolder syntheses that translate mechanistic insight into an extended health span.
